# Beyond the liver: a case of solitary skull lesion with hepatocellular features without a primary malignancy

**DOI:** 10.3332/ecancer.2025.1961

**Published:** 2025-08-07

**Authors:** Asma Saleem Khan, Quratulain Badar, Muhammad Nauman Zahir, Kaynat Siddiqui

**Affiliations:** 1 Department of Radiation Oncology, Dr. Ziauddin Hospital, Karachi 74700, Pakistan; 2 Department of Medical Oncology, Dr. Ziauddin Hospital, Karachi 74700, Pakistan

**Keywords:** hepatocellular carcinoma, hepatoid adenocarcinoma, solitary bone lesion, unknown primary

## Abstract

Hepatocellular carcinoma (HCC) is primarily a malignant liver tumour. In rare cases, HCC may be asymptomatic and incidentally identified in radiological exams. Although extrahepatic metastasis to organs such as the lungs or bones is not very uncommon, isolated extrahepatic lesions with hepatoid characteristics, in the absence of a primary tumour in the liver or gastrointestinal tract, are incredibly uncommon. Herein, we present an intriguing case of an isolated skull lesion with hepatoid features with no identifiable primary hepatic or gastrointestinal lesion. This case highlights the need for further research into these rare presentations to deepen our understanding of HCC pathogenesis and ectopic tissue transformation.

## Introduction

Hepatocellular carcinoma (HCC) is the most common and violent primary liver malignancy with a poor prognosis when diagnosed at advanced stages [1]. It typically presents at later stages with a variety of specific and nonspecific symptoms, but uncommonly it is identified incidentally during radiological imaging [2].

Although HCC often metastasises to other sites, including the regional lymph nodes, lungs and bones, cases with an HCC-like extrahepatic lesion without detection of a primary liver or gastrointestinal lesion are exceptionally rare and pose a diagnostic dilemma. Such cases suggest the possibility of ectopic liver tissue or hepatoid differentiation in other tissues, an unusual phenomenon that has been sporadically documented but is not well understood.

We present a rare case of an isolated skull lesion with hepatoid features, without any detectable primary liver or gastrointestinal tumour. This case underscores the importance of recognising atypical HCC-like presentations and provides insights into hepatoid differentiation and ectopic tissue transformation, expanding our understanding of hepatocellular pathology beyond the liver.

## Case presentation

A 67-year-old male presented with a 3-month history of progressively enlarging right temporoparietal swelling. He had no other symptoms. Examination showed a Karnofsky Performance Status of 100, with slight difficulty walking and mild weakness (4/5) in the left proximal lower limb, while other limbs had normal strength.

**Figure 1.**
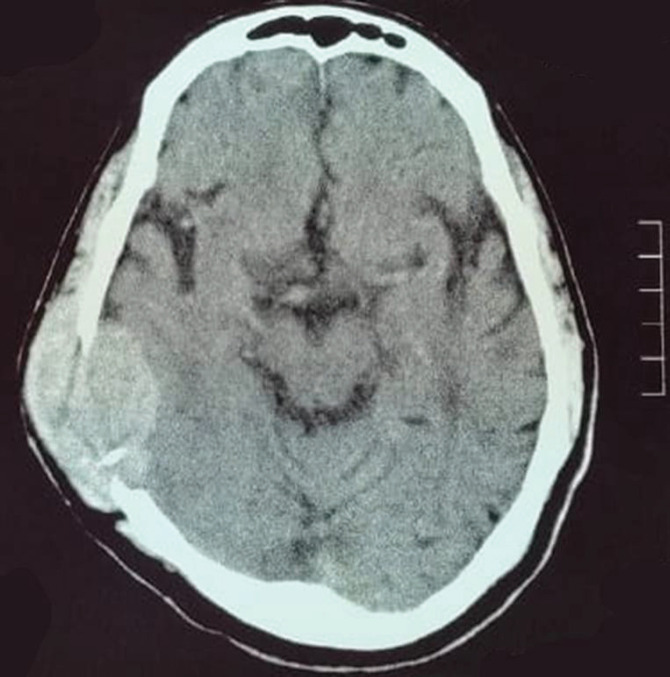
CT brain axial slice shows soft tissue mass within the right temporoparietal bone causing bone destruction, and compression of adjacent cortical sulci.

**Figure 2.**
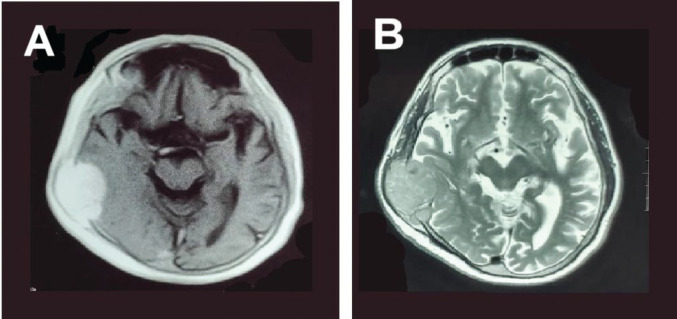
MRI brain scan with contrast, axial slices (a) T1-weighted and (b) T2-weighted images showing a homogenous well defined, isointense lesion in the right temporoparietal region causing compression and destruction of the cortex of the temporal and parietal bone.

A brain computed tomography (CT) scan (Figure 1) identified a soft tissue mass in the right temporoparietal bone, causing local bone destruction and extending into the epidural space with compression of the cortical sulci. The lesion measured approximately 4.9 × 3.5 cm. Brain magnetic resonance imaging (MRI) with contrast (Figure 2a) revealed a hyper-intense, extra-axial mass with bone erosion extending into the scalp and compressing adjacent cortical sulci, measuring 4.9 × 3.3 × 4.5 cm and accompanied by edema in the right temporal lobe. Differential diagnoses included hemangiopericytoma and atypical meningioma. An additional MRI study (Figure 2b) further characterised the mass as destructive to the right parietal and temporal bones with some bone marrow infiltration, though no significant brain parenchyma edema was observed.

**Figure 3.**
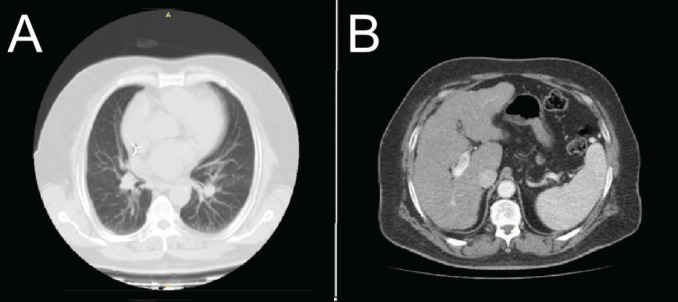
CT chest and abdomen scan with contrast, axial slices (a): Lung window shows no focal pleuropulmonary lesion; (b): Abdomen shows irregular liver borders with no focal mass lesion.

CT imaging of the chest and abdomen (Figure 3a and b) showed no pleuropulmonary or abdominal metastasis.

**Figure 4.**
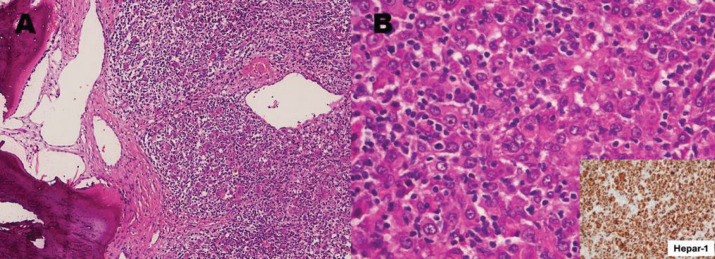
Right temporal mass excisional biopsy. (a): Exhibiting bone and soft tissue involved by malignant poorly differentiated neoplasm which based on diffuse immunohistochemical expression of cytokeratin metastatic carcinoma. (b): Polygonal cells with abundant eosinophilic cytoplasm with diffuse immunohistochemical expression of Hepar-1 stain (inset) consistent with hepatoid differentiation, raising a differential diagnosis of liver origin (HCC) versus HAC from the upper gastrointestinal tract (H&E, 20×).

The patient underwent a right temporoparietal craniotomy with lesion excision and cranioplasty. Histopathology showed large polygonal cells with eosinophilic cytoplasm, hyperchromasia and intranuclear inclusions, consistent with metastatic carcinoma. Immunohistochemistry (IHC) was positive for CK AE1/AE3, HepPar1 and Arginase, suggesting a primary liver origin, while CD34 was negative in tumour cells but highlighted vascular structures (Figure 4a and b). Differentials included HCC and hepatoid adenocarcinoma (HAC) of upper gastrointestinal origin.

The case was discussed in the multidisciplinary tumour board meeting, where it was recommended for further testing. Serum Alpha-­Fetoprotein (AFP) was 6.7 ng/mL, HBsAg and anti-HCV tests were negative and PIVKA-II was mildly elevated at 107.05 mAU/mL.

**Figure 5.**
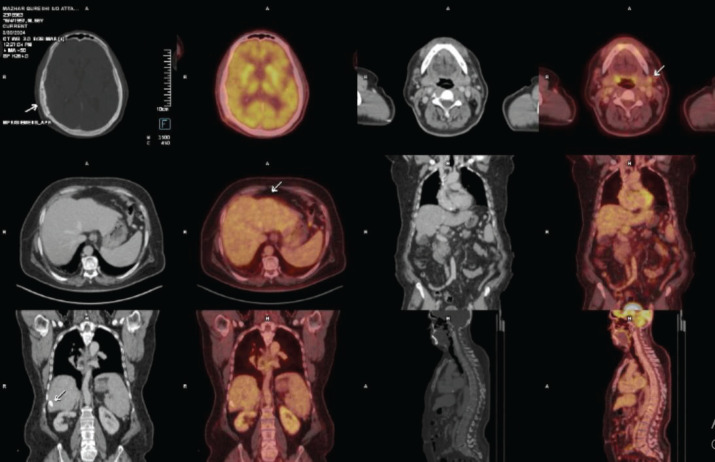
PET images showing post-craniotomy changes in the skull bone as represented by the 1st arrow. The second arrow shows the reactive cervical node, while the third and fourth arrows represent the non-avid focal area of calcifications within the liver.

The positron emission tomography (PET)/CT scan revealed post-surgical changes but no Fluorodeoxyglucose (FDG)-avid lesions at the surgical site. A small FDG-avid left cervical lymph node indicated possible inflammation. The liver exhibited signs of chronic disease, with nodular margins and two calcified hypodense areas in segment VI, likely benign (Figure 5).

The patient was referred to the radiation oncology department, and the skull lesion was treated with radiation therapy, 30 Gy in 10 fractions, to manage symptoms and improve quality of life. The patient was kept under close surveillance by US abdomen every 3 months and CT Abdomen and pelvis, PET/CT every 6 months. The patient had a recent follow-up with a PET CT scan, which showed similar findings as previously reported. No FDG-avid lesion was appreciated.

## Discussion and conclusion

HCC is primarily a liver malignancy. According to the GLOBOCAN 2022, it ranks third most common cause of cancer-related mortality worldwide. Due to its violent nature, the overall 5-year survival is only 18 percent [3]. This carcinoma metastasises to regional lymph nodes, lungs, bones and adrenal glands [4]. Due to the recent advancements in radiological techniques, a large number of cases of HCC with bone metastases are reported now [5]. The most frequent skeletal sites are the vertebral column, pelvic bones and ribs. Although the skull is a very uncommon site for HCC metastases, it is more notably documented in the recent literature [4].

HAC is rather an infrequent and challenging malignancy, distinguished histologically by its similarity to HCC. HAC, compared to other adenocarcinomas, is different in its clinical course and biological behaviour. HAC can develop in various organs, including the stomach, esophagus, colon, pancreas, lungs and ovaries. This multi-organ potential leads to difficulty in its diagnosis and treatment [6]. In our case, the IHC was diffusely positive for arginase-1 and HePar1, which distinguished it from HAC.

In rare cases of HCC, patients present with solitary bone lesions with no detectable primary liver tumour [7, 8]. Takahama *et al* [5] reported an iliac bone lesion, initially diagnosed due to high serum AFP and PIVKA II levels, alongside IHC staining that confirmed HCC despite no primary liver involvement. Similarly, Guo *et al* [4] reported a case where a painless skull lesion eventually revealed a large underlying liver mass, confirming HCC. In our case, the patient presented with a painless skull swelling in the temporoparietal region, with no detectable primary liver lesion. Serum AFP levels were normal, while PIVKA II was mildly elevated. However, IHC staining indicated a hepatic origin despite the absence of a focal liver or gastrointestinal mass on PET/CT imaging. AFP, though commonly used as an HCC biomarker, has low sensitivity and specificity. AFP levels above 400 ng/mL often suggest HCC, but only some patients show such elevations, limiting its diagnostic reliability, especially in early HCC. Therefore, combining additional biomarkers like PIVKA II with imaging and IHC staining enhances diagnostic accuracy [9].

The data for a solitary bone lesion favouring hepatic origin with no primary lesion is scarce [7, 8]. A solitary skull lesion with pathologically proven carcinoma of hepatic origin and no lesion in the liver has not been reported previously in the literature.

Our findings support the hypothesis that bone marrow may serve as a potential origin for HCC. Studies suggest that bone marrow stem cells can migrate to the liver and differentiate into hepatocytes or HCC cells. Additionally, the cytokine-rich environment within bone marrow may encourage mesenchymal cell differentiation into hepatocytes, providing a supportive niche for HCC development, particularly in the spine [10].

Several treatment options can be used to treat skull metastases from HCC, including direct ethanol injection therapy, radiotherapy, surgical resection and supportive management [11]. In our case, the patient was treated with radiotherapy. The patient was then kept under close surveillance with a PET/CT scan and no abnormality to suggest a primary disease was seen at 12-month follow-ups.

Our case of a solitary skull lesion without a detectable primary liver mass supports the hypothesis of an extrahepatic HCC origin within the bone marrow. The concept of ectopic liver tissue forming in the bone marrow, although undocumented, warrants further investigation. Recurrence-free survival observed with local treatment in similar cases suggests these tumours might be primary lesions rather than metastases. This evidence bolsters the theory of bone marrow as a potential site for primary HCC formation, highlighting a promising area for further research.

## List of abbreviations

AFP, Serum alpha-fetoprotein; HAC, Hepatoid adenocarcinoma; HCC, Hepatocellular carcinoma; PET, Positron emission tomography; PIVKA-II, Protein induced by vitamin K absence-II.

## Conflicts of interest

All authors declare no conflicts of interest.

## Funding

The authors declare no funding support for the writing of this article.

## Ethics statement

This case was presented at Dr Ziauddin University Hospital, Karachi, Pakistan. Ethical approval was not required for this case report, as confirmed by the Ethics Review Committee (ERC) following institutional guidelines for single-patient case reporting.

## Informed consent

An informed written consent was obtained from the patient for the publication of this case report.

## Trial registration

This article is not registered for any clinical trial.

## Author contributions

Asma Saleem Khan: Substantial contributions to concept or design, acquisition, analysis or interpretation of data, drafting of the manuscript, critical review of the manuscript for important intellectual content, agreed to be accountable for all aspects of the work, reviewed the final version to be published.

Quratulain Badar: Critical review of the manuscript for important intellectual content, agreed to be accountable for all aspects of the work, reviewed the final version to be published and supervised the work.

Muhammad Nauman Zahir: Critical review of the manuscript for important intellectual content, agreed to be accountable for all aspects of the work, reviewed the final version to be published and supervised the work.

Kaynat Siddiqui: Critical review of the manuscript for important intellectual content, agreed to be accountable for all aspects of the work, reviewed the final version to be published.

Zeeshan Uddin: Critical review of the manuscript for important intellectual content, agreed to be accountable for all aspects of the work, reviewed the final version to be published.
